# Supplementary material for: Self-, other-, and meta-perceptions of personality: Relations with burnout symptoms and eudaimonic workplace well-being
Source: PLoS One. 2022 Jul 28;17(7):e0272095. doi: 10.1371/journal.pone.0272095 (PMC9333331; doi:10.1371/journal.pone.0272095)
Supplement: S1 Text — (DOCX) [file pone.0272095.s001.docx]

S1 Text. Data checks.

Data quality checks

##### In total, 435 participants and 963 of their colleagues (who provided other-ratings of personality) participated in this study. After the data collection, participant responses were screened based on three data quality checks. First, we checked whether respondents overused the same response option (i.e., showed very little variation in use of response options). In line with Lee and Ashton’s advice [1], [see also 2], we computed the standard deviation of the responses on all HEXACO-96 items (see measurements), *before* recoding of reverse-keyed items, using a critical value of .70. Eighteen informant reports were excluded based on this criterion.

##### Second, to screen out respondents who provided extremely incoherent responses, we computed a standard deviation of the item responses on each of the six HEXACO factor-level scales (*after* recoding of reverse-keyed items). For each respondent, an average of the six standard deviations was calculated. According to Lee and Ashton [1], an average higher than 1.60 is highly unlikely. One respondent was excluded based on this quality check (including two corresponding informant reports).

##### Third, we assessed the quality of the obtained informant personality ratings. There were 36 participants (8.3%) who did not provide any informant personality rating. These participants were excluded from further analyses. Furthermore, 39 participants (9%) provided only one informant personality rating, 173 participants (39.9%) provided two and 186 participants (42.8%) provided three informant personality ratings. In order to measure reliability of the informant reports, the intraclass correlation coefficient (ICC) was computed for each of the six HEXACO factor-level scales (results are shown in S1 Table). The resulting ICC gives a reliability estimate based on the degree of inter-rater agreement. ICC estimates and the 95% confident intervals are based on absolute-agreement, one-way random effects model. For the calculation of the ICC, the intended measurement protocol was taken into account (for details, see [3]). The intended measurement protocol states whether the other-rating of personality is based on one informant report (ICC model 1) or on the mean of two or three informant reports (ICC model 2 and 3 respectively). An ICC of <.40 was considered as *poor*, a range of .40–.59 as *fair*, a range of .60–.74 as *good*, and an ICC of >.75 was considered as *excellent* [4]. When the intended measurement protocol was set to one informant report, the reliability was poor for Honesty-Humility (i.e., .29). When the intended measurement protocol was set to the average of two, the inter-rater reliability for each of the six factor-level scales was acceptable, ranging from .45 for Honesty-Humility to .73 for Openness to Experience. Therefore, all cases with only one informant personality rating were excluded (*N* = 39). Subsequently, for each participant, averaged informant report scores (based on two or three informants) were determined for each personality factor scale. After the data quality checks and subsequent exclusion of respondents, the final sample included 359 participants, providing 906 informant reports.

Data check percentages of in agreement values and discrepant values

We checked the data to ensure the presence of sufficient discrepancies between personality ratings (10% or more in either direction), as this is a prerequisite for any further analyses examining agreement and discrepancy [5]. Therefore, standardized scores were calculated for each personality factor scale [6]. As mentioned in Shanock et al. [5], a standardized score on one variable (e.g., self-reported Extraversion) that differs half a standard deviation or more from another variable (e.g., averaged others’ reported Extraversion) is considered as discrepant. We determined the percentages of in agreement values and the percentages of discrepant values in either direction. S4 Table shows that the percentage of discrepancies between personality ratings in either direction exceeds the required 10% for each personality factor scale. This means the data is suitable to explore our question whether (dis)agreement between personality perceptions relates to burnout and workplace well-being within a polynomial regression model.

References

1. Lee K, Ashton MC. Psychometric properties of the HEXACO-100. Assessment. 2018;25(5):543-556. doi: 10.1177/1073191116659134

2. Barends AJ, De Vries RE. Noncompliant responding: Comparing exclusion criteria in MTurk personality research to improve data quality. Personality and Individual Differences. 2019;143:84-89. doi: 10.1016/j.paid.2019.02.015

3. Koo TK, Li MY. A Guideline of Selecting and Reporting Intraclass Correlation Coefficients for Reliability Research. Journal of Chiropractic Medicine. 2016;15(2):155-163. doi: 10.1016/j.jcm.2016.02.012

4. Cicchetti DV. Guidelines, criteria, and rules of thumb for evaluating normed and standardized assessment instruments in psychology. Psychological Assessment. 1994;6(4):284-290. doi: 10.1037/1040-3590.6.4.284

5. Shanock LR, Baran BE, Gentry WA, Pattison SC, Heggestad ED. Polynomial regression with response surface analysis: A powerful approach for examining moderation and overcoming limitations of difference scores. Journal of Business and Psychology. 2010;25(4):543-554. doi: 10.1007/s10869-010-9183-4

6. Fleenor JW, McCauley CD, Brutus S. Self-other rating agreement and leader effectiveness. The Leadership Quarterly. 1996;7(4):487-506. doi: 10.1016/s1048-9843(96)90003-x
