# Supplementary figures and images for: Self-, other-, and meta-perceptions of personality: Relations with burnout symptoms and eudaimonic workplace well-being
Source: PLoS One. 2022 Jul 28;17(7):e0272095. doi: 10.1371/journal.pone.0272095 (PMC9333331; doi:10.1371/journal.pone.0272095)

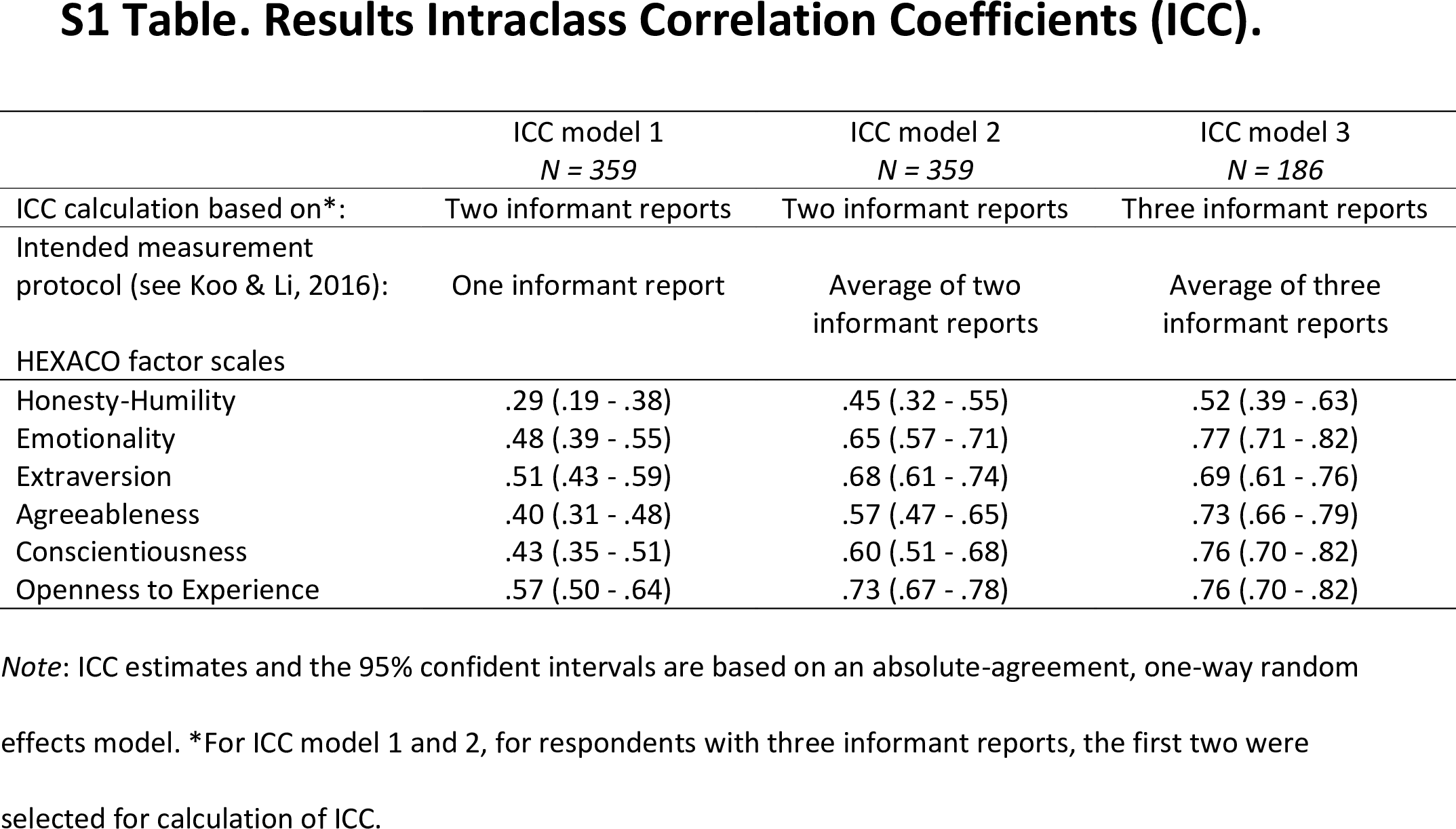

Supplement: S1 Table — (TIF) [file pone.0272095.s002.tif]

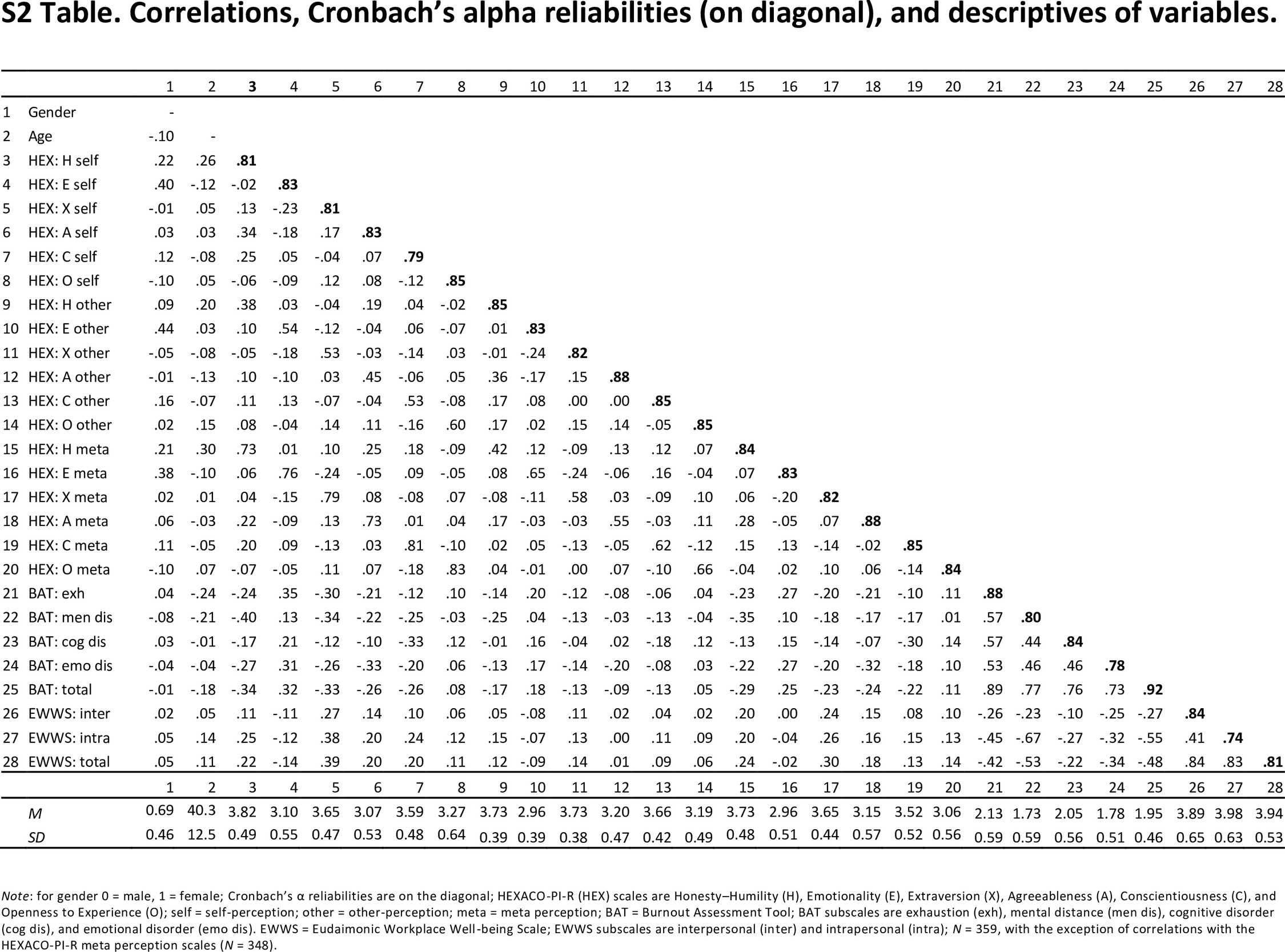

Supplement: S2 Table — (TIF) [file pone.0272095.s003.tif]

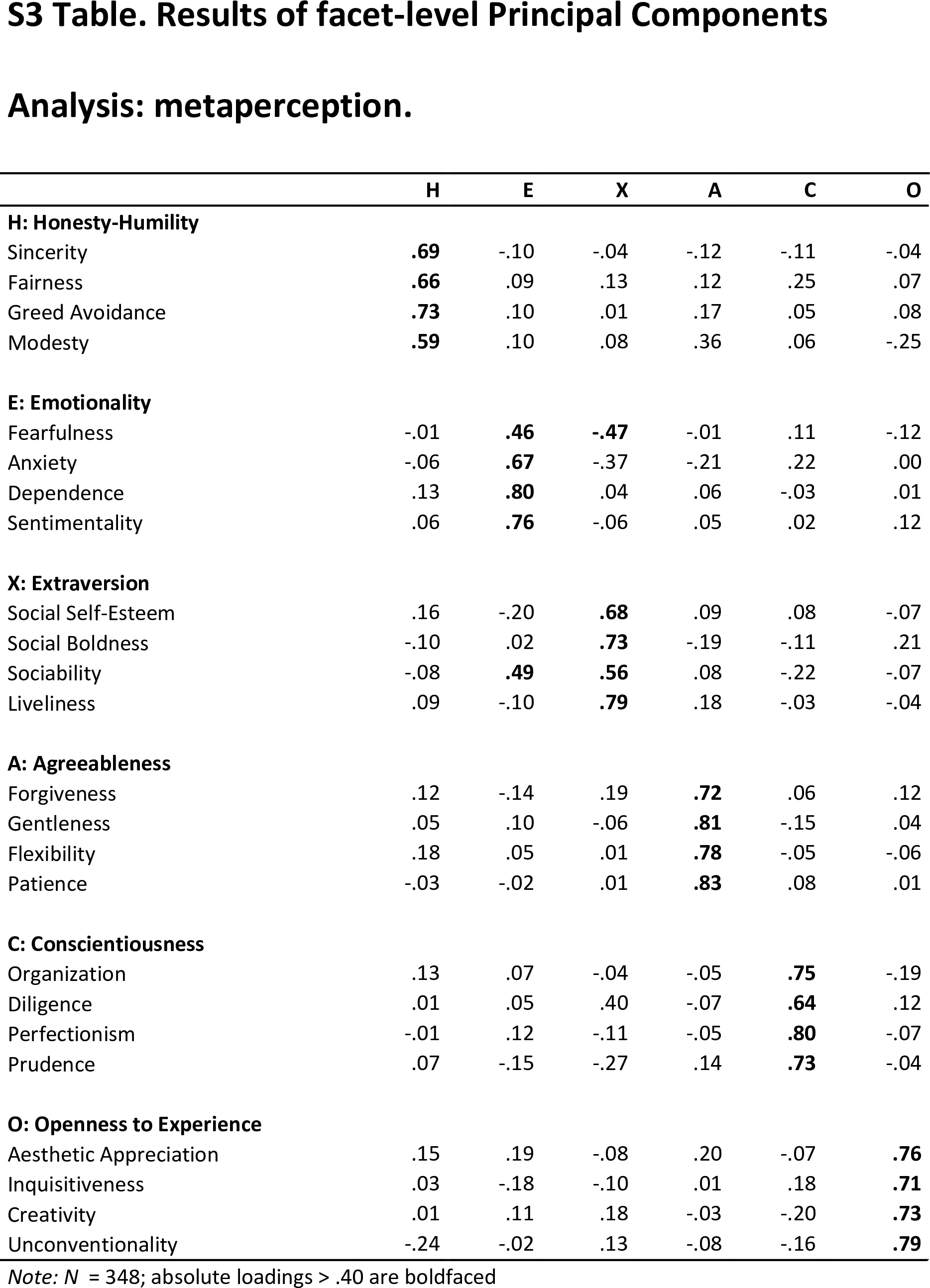

Supplement: S3 Table — (TIF) [file pone.0272095.s004.tif]

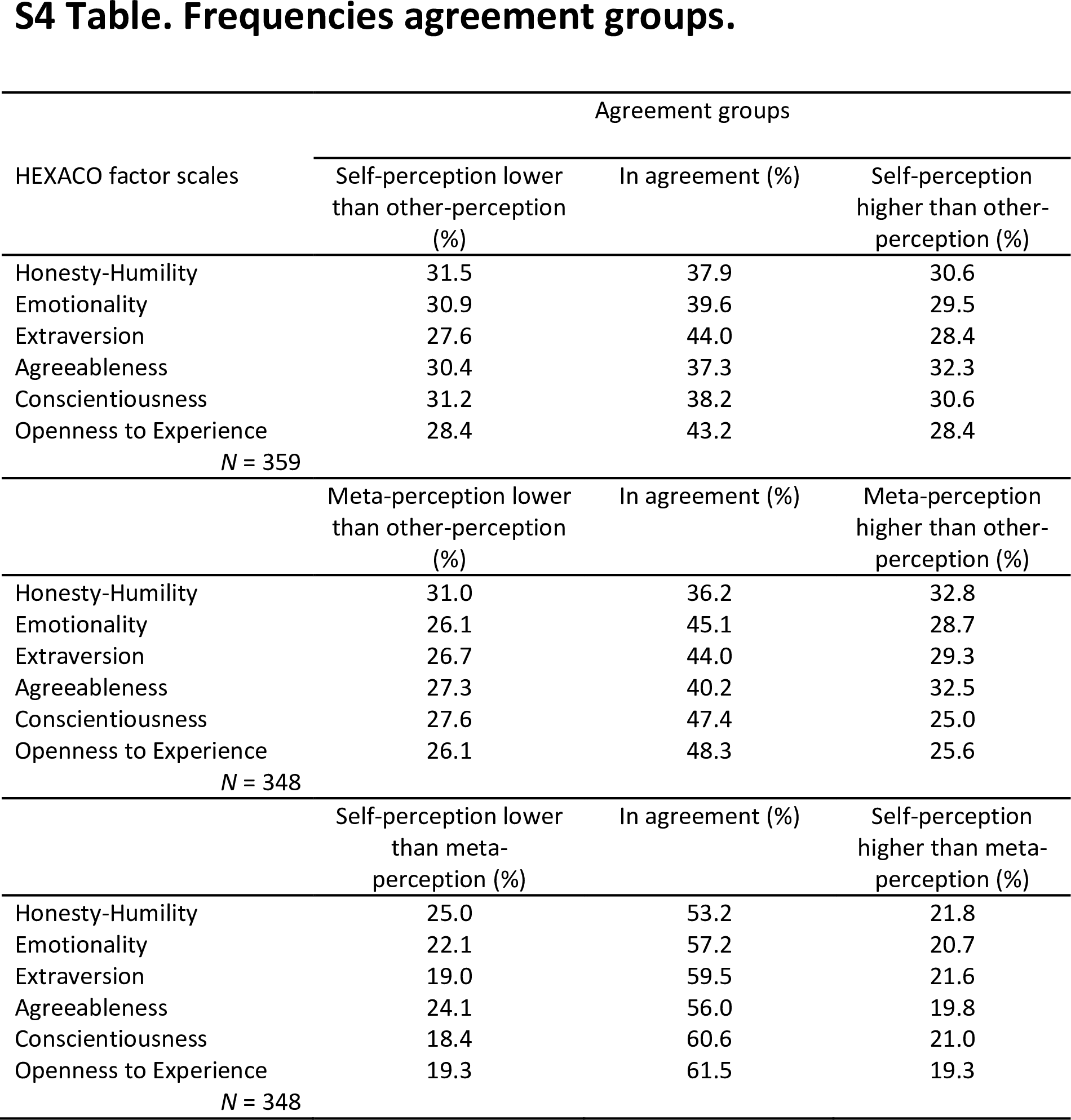

Supplement: S4 Table — (TIF) [file pone.0272095.s005.tif]

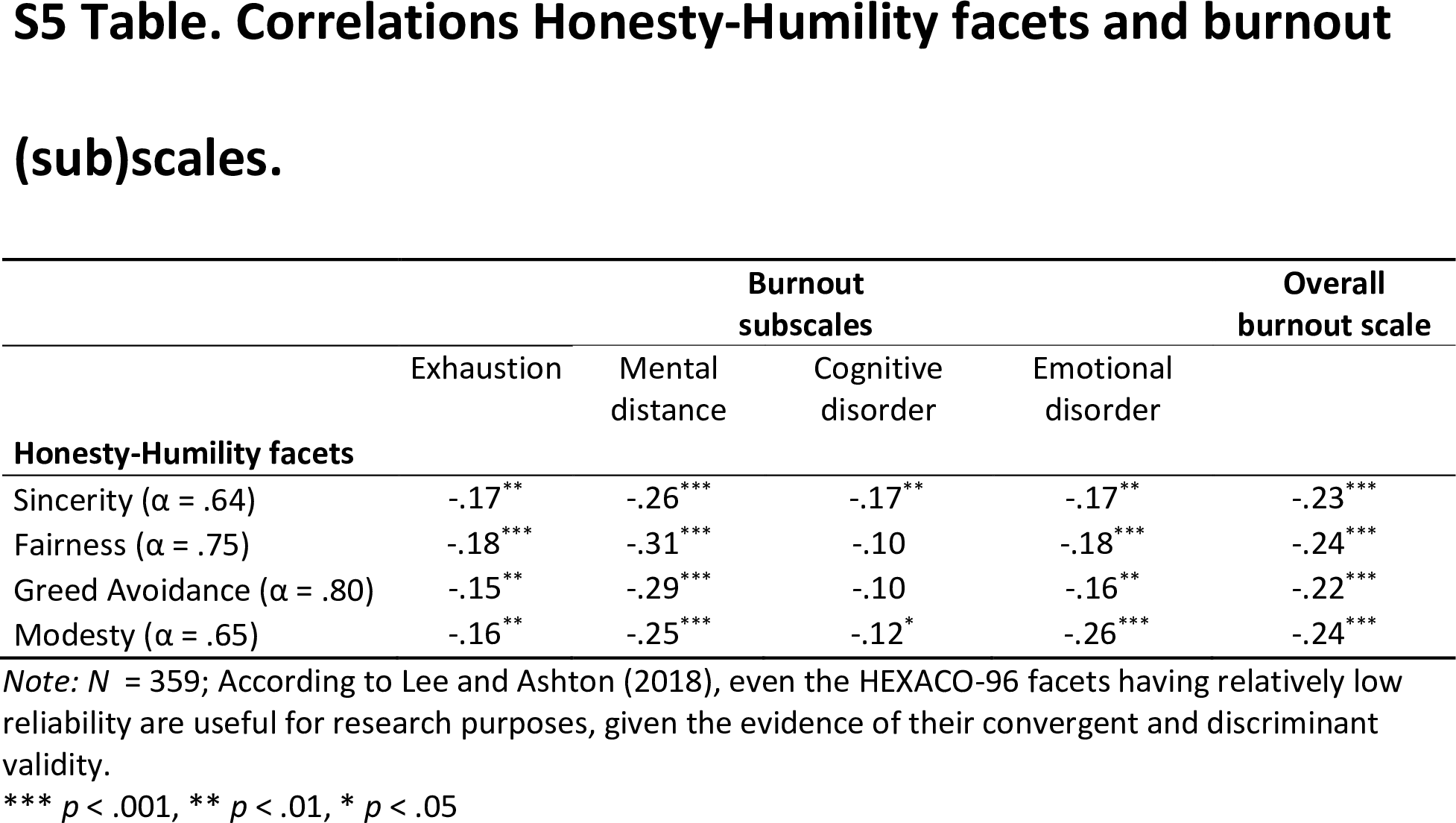

Supplement: S5 Table — (TIF) [file pone.0272095.s006.tif]
