# Supplementary material for: Self-, other-, and meta-perceptions of personality: Relations with burnout symptoms and eudaimonic workplace well-being
Source: PLoS One. 2022 Jul 28;17(7):e0272095. doi: 10.1371/journal.pone.0272095 (PMC9333331; doi:10.1371/journal.pone.0272095)
Supplement: Attachment — Submitted filename: Response to reviewer(s) new.docx [file pone.0272095.s008.docx]

Amsterdam, 28^th^ of June, 2022,

Dear editor and reviewer(s),

Thank you for the opportunity to revise and resubmit our manuscript again. In this letter, we explain how we have addressed the issue concerning point 3 page 3, data accessibility.

We hope that this new draft meets your expectations and that of the reviewer(s) for publication in *PLOSONE*.

We are again looking forward to your response.

Sincerely,

on behalf of all the authors

Wim Bloemers, corresponding author

**Points raised by the academic editor:**

1) S2 Table: please add a top row with the variable number for easier reference.

*We added a top row.*

2) S5 Table: please also include the overall burnout score in line with BAT's presentation that burnout should also be considered an overall score

*We added correlations between the facets of Honesty-Humility and the overall burnout score.*

**Journal Requirements:**

1) Please ensure that your manuscript meets PLOS ONE's style requirements, including those for file naming.

*To the best of our knowledge, the manuscript is in line with the style requirements.*

2) Please provide additional details regarding participant consent. In the ethics statement in the Methods and online submission information, please ensure that you have specified (1) whether consent was informed and (2) what type you obtained (for instance, written or verbal, and if verbal, how it was documented and witnessed). If the need for consent was waived by the ethics committee, please include this information.

*In the methods section, we added additional details regarding participant consent. In the original manuscript we already included information about the ethical committee:*

*“In the period from November 2019 to March 2020, 15 student members of a research team at the Open University in the Netherlands collected data for the current study. To obtain a diverse sample, the students approached potential participants in their own networks (family, friends, and colleagues). Information letters were used for recruitment, stating that participants (minimum age: 18 years) were required to be presently employed and to have at least three colleagues with whom they often collaborated. Potential participants were asked to complete (personality) questionnaires at two different moments and to obtain personality ratings from three close colleagues. The information letter provided detailed information about the study and emphasized that participation was voluntary, confidential, and could be stopped at any time. In addition, it was clearly stated that participants did not have access to the answers of their colleagues, and vice versa. The information letter contained a link to the first online questionnaire (T1) and started with an informed consent question, followed by background variables, a self-perception personality inventory, and questions measuring work-related burnout symptoms and eudaimonic workplace well-being. Next, participants received a second information letter, asking them to approach three colleagues for informant reports on the personality inventory (T2). This second letter for colleagues also provided detailed information about the study and a link to the online questionnaire, which started with an informed consent question. One week after having finished the T1-questionnaire, participants received an e-mail with access to the meta-perception personality inventory (T3). In exchange for participation, respondents were offered individualized personality reports (based on their self-perception). The entire procedure of data collection was approved by the Ethical Committee of the Open University of the Netherlands (correspondence November 5, 2019, registration number: U/2019/08651).”*

3) In your revised cover letter, please address the following prompts:

a) If there are ethical or legal restrictions on sharing a de-identified data set, please explain them in detail (e.g., data contain potentially sensitive information, data are owned by a third-party organization, etc.) and who has imposed them (e.g., an ethics committee). Please also provide contact information for a data access committee, ethics committee, or other institutional body to which data requests may be sent.

b) If there are no restrictions, please upload the minimal anonymized data set necessary to replicate your study findings as either Supporting Information files or to a stable, public repository and provide us with the relevant URLs, DOIs, or accession numbers. For a list of acceptable repositories, please see <http://journals.plos.org/plosone/s/data-availability#loc-recommended-repositories>

a) Our data can be accessed through the data steward of the Open University of the Netherlands, Dr. Mellanie Geijsen, email: [datasteward@ou.nl](mailto:datasteward@ou.nl). For further information you can contact the Ethics committee: [ceto@ou.nl](mailto:ceto@ou.nl).
b) There are no ethical or legal restrictions on sharing a de-identified data set. We uploaded our anonymized data set with our other revised materials: *data file metapercep Pone-D-21-35155.sav.*
4) We note that you have referenced (Schaufeli WB, De Witte et al. [15]) which has currently not yet been accepted for publication. Please remove this from your References and amend this to state in the body of your manuscript: (Schaufeli WB, De Witte et al. [15 [Unpublished]”) as detailed online in our guide for authors.

*In the meantime it has been accepted for publication, so we changed the reference into:*

*Schaufeli WB, Desart S, De Witte H. Burnout Assessment Tool (BAT)—Development, Validity, and Reliability. International Journal of Environmental Research and Public Health. 2020; 17(24):9495. https://doi.org/10.3390/ijerph17249495*

**Point raised by the academic editor and reviewer(s):**

1) Can you not be more specific in the abstract, given the results inside the article itself:

"Only when respondents misjudged how their colleagues would rate them on Honesty-Humility (i.e., discrepancy between meta- and other-perceptions), respondents experienced more feelings of burnout."

"Overall, our results provide evidence that discrepancies between meta- and other-perceptions of Honesty-Humility affect employee well-being (i.e., burnout and eudaimonic workplace well-being)."

*We changed the end of the abstract in order to be more specific about the most important results. “The present study examined whether disagreement between self-, other-, and meta-perceptions of personality was related to burnout symptoms and eudaimonic workplace well-being. We expected disagreement in personality perceptions to explain incremental variance in burnout symptoms and eudaimonic workplace well-being beyond the main effects of the different personality ratings. Participants were 459 Dutch employees and their 906 colleagues (who provided other ratings of personality). The results, based on polynomial regression with response surface analyses, highlighted strong main effects of self-rated personality traits in relation to burnout symptoms and eudaimonic workplace well-being. This study provides, as far as we know, the first empirical evidence that self-rated Honesty-Humility negatively predicts burnout symptoms. Results showed little evidence on incremental effects of disagreement between personality perceptions, with one clear exception: when respondents misjudged how their colleagues would rate them on Honesty-Humility (i.e., discrepancy between meta- and other-perceptions), respondents experienced more feelings of burnout and less eudaimonic workplace well-being. Our study contributes to the literature by providing evidence that discrepancies between meta- and other-perceptions of Honesty-Humility affect employee well-being (i.e., burnout symptoms and eudaimonic workplace well-being).”*

2) Is the sentence in 505-508 correct? It reads strange to me. I assume the "this" should be "his". Furthermore, I am not sure if this "understanding" explanation here is necessarily accurate. Could it not be that because less conscientious that they do not feel as much pressure in the workplace? Just a thought.

*Reviewer rightly indicted a writing error in line 505. We changed ‘this’ into ‘his’.*

*Furthermore, we thank the reviewer for his/her suggestion and added: “Agreement on being less conscientious could also imply experiencing lower work pressure (e.g., accepting that somebody will do less work).”*

3) Line 606. I think the caveat should just be added that selecting only on one trait should of course be done with caution, but that it does seem to be a protective factor according to your results.

*We added: “Even though selection should not be based solely on applicant questionnaire scores on Honesty-Humility, it can be taken into account since it seems to be a protective factor. The Honesty-Humility scores and its potential protective function could for example be discussed with applicants in a post questionnaire interview.”*

4) Lines 608+ is a very interesting and practical suggestion. I know some consultants present team development workshops for organizations that help employees understand each other better. Perhaps you can state it (somewhat) as blatantly as that.

*Many thanks for your input on team development workshops. We added the suggestion about organizing such workshops in order to understand each other better in the manuscript on pages 29-30.*

5) Is there any research on honesty-humility and depression? The reason I am asking this is because of the criticism that burnout as a concept is receiving (that it is just depression) if your results don't perhaps reveal something interesting compared to past findings. If there is something a short addition at the appropriate instance in the manuscript might be useful. This is just a strong suggestion, I don't want to force it on you. But if there is something, as I said, this would be interesting in the context of your results.

*We did not find research on the relation between Honesty-Humility and depression, but we did find previous literature on Honesty-Humility as a possible moderator of the relation between job demands and exhaustion. So, we added two references:*

*“Although Honesty-Humility previously has been identified as a possible moderator of the relation between job demands and exhaustion [65, 66], this study provided, as far as we know, the first empirical evidence that Honesty-Humility negatively predicts burnout symptoms.”*

*65. Ceschi A, Sartori R, Dickert S, Costantini A. Grit or honesty-humility? New insights into the moderating role of personality between the health impairment process and counterproductive work behavior. Frontiers in Psychology. 2016;7:1799. doi:10.3389/fpsyg.2016.01799*

*66. Toussaint LL, Webb JR. A theoretical model and review of evidence linking humility to health and well-being. In: Handbook of humility: Theory, research, and applications. 2016.*

6) To me it is not entirely clear how you constructed your burnout score. I think it is important to state how you constructed it from its 4 components. I was also unable to see the supplementary material only the main manuscript.

*We added (see measurements, burnout symptoms): “Therefore, based on the unit-weighted combination of the four subscales, one composite score was made as a general indicator for burnout symptoms.”*

7) Be sure to submit high quality images for the Figures that will show well in a PDF document. I found it hard to read some of them.

*We adjusted the resolution of the figure and hope it is now easy to read.*

8) "alpha reliabilities" add Cronbach's before alpha.

*We added Cronbach’s in all relevant places.*

As far as we can see we have revised our manuscript, figures and tables in full compliance with the reviewer(s) remarks and suggestions. We also added the SPSS data file data file *meta perception with dummy email addresses DEF.sav*.

Awaiting your final answer on our manuscript,

On behalf of all the authors,

Yours sincerely,

Wim Bloemers

Corresponding author
